# Negative and depressive symptomatology in dual psychosis: three-month follow-up comparative analysis

**DOI:** 10.1192/j.eurpsy.2025.670

**Published:** 2025-08-26

**Authors:** C. Rusen, R. Gimeno, J. Renau, M. Cabañes, A. Moreno, M. Peraire

**Affiliations:** 1 Department of Mental Health, Consorcio Hospitalario Provincial Castellon; 2 Universidad Cardenal Herrera-CEU Universities, TXP Research Group, Castellon, Spain

## Abstract

**Introduction:**

Within the range of psychotic symptoms, negative symptomatology is the one that presents the least effective treatments. Depressive symptoms share characteristics with this symptomatology, making it difficult to distinguish between the two (Edwards *et al.* Psychol.Med. 2019; 1-13). In daily practice, antidepressants are used to treat the negative symptoms of psychosis, due to the absence of therapeutic alternatives.

**Objectives:**

The aim of this work is to analyse the correlation between these symptomatic groups, comparing the effectiveness of treatment with antidepressants during a three-month follow-up.

**Methods:**

Longitudinal and prospective clinical trial on a sample of 127 patients (95 men, 32 women) with dual psychosis (85 with schizophrenia, 42 with schizoaffective disorder), recruited from community or hospital facilities of the Hospital Provincial of Castellon. The mean age was 38.5 years. Antidepressant prescription was analysed at 4 different times, each month during follow-up, with PANSS-N for negative symptoms and MADRS and CDSS for depressive symptomatology.

Psychometric comparisons were made between the intervention group (16 patients with prescribed antidepressants, in therapeutic doses during the three months) and the control group (111 patients).

**Results:**

Regarding the correlation between the 3 scales, we observed that at T0 there is no significant correlation, while at T1, T2 and T3 this correlation is significant (p<0.01), as the scores improve. A progressive increase in Pearson’s coefficient (PCC) is observed between T1 and T3, emphasizing a higher correlation between MADRS and CDSS (PCC of 0.920 at T3) with respect to PANSS-N and the other two questionnaires (PCC of 0.587 and 0.619 at T3, respectively).

Comparing the means between the scales, there is a significant decrease in MADRS between T0 and T3 (61.8% decrease from baseline, F: 4.49, p<0.05), as well as in CDSS (-68.7%, F: 4.53, p<0.05). In PANSS-N there are no significant differences (F: 0.57, p: 0.45), despite a relative decrease of 51.9%.

Considering clozapine prescription, there is a significant decrease in MADRS and CDSS during the first month, with no differences in PANSS-N throughout the evolution.

**Conclusions:**

The reduction obtained in the MADRS and CDSS scales can be associated with the prescription of antidepressants, as opposite to the reduction obtained in PANSS-N. This implies that antidepressants do not influence the reduction in negative symptoms, reflecting the clinical impression that the two entities are distinct (despite areas of overlap).

The analysis using clozapine treatment reinforces the existence of a different idiosyncrasy between symptomatic groups. This can be explained at the neurobiological level by the different mechanisms of action involved (monoamine depletion vs. dopaminergic blockade), but keeps the debate open as to how they can be differentiated and treated in clinical practice.

**Disclosure of Interest:**

None Declared

